# Examining alignment of community health teams' preferences for health, equity, and spending with state all‐payer waiver priorities: A discrete choice experiment

**DOI:** 10.1111/1475-6773.14257

**Published:** 2023-11-14

**Authors:** Eline M. van den Broek‐Altenburg, Jamie S. Benson, Adam J. Atherly

**Affiliations:** ^1^ Larner College of Medicine University of Vermont Burlington Vermont USA; ^2^ Perelman School of Medicine University of Pennsylvania Philadelphia Pennsylvania USA; ^3^ College of Health Professions Virginia Commonwealth University Richmond Virginia USA

**Keywords:** healthcare organizations and systems, program evaluation, sampling, social work, survey research and questionnaire design

## Abstract

**Objective:**

The state of Vermont has a statewide waiver from the centers for medicare and medicaid services to allow all‐payer Accountable Care Organizations (ACOs). The Vermont all‐payer model (VAPM) waiver is layered upon previous reforms establishing regional community health teams (CHTs) and medical homes. The waiver is intended to incentivize healthcare value and quality and create alignment between health system payers, providers, and CHTs. The objective of this study was to examine CHT's trade‐offs and preferences for health, equity, and spending and the alignment with VAPM priorities.

**Data Sources/Study Setting:**

Data were gathered from a survey and discrete choice experiment among CHT leadership and CHT team members of the 13 CHTs in Vermont.

**Study Design:**

We used conditional logit models to model the choice as a function of its characteristics (attributes) and mixed logit models to analyze whether preferences for programs varied by persons and roles within CHTs.

**Data Collection/Extraction Methods:**

There were 60 respondents who completed the survey online with 14 choice tasks, with three program options in each task, for a total sample size of 2520.

**Principal Findings:**

We found that CHTs prioritized programs in the community health plan and those with quantitative evidence of effectiveness. They were less likely to choose either programs targeting racial and ethnic minorities or programs having a small effect on a large population. Preferences did not vary across individual or community attributes. Program priorities of the VAPM, especially healthcare spending, were not prioritized.

**Conclusions:**

The results suggest that the new VAPM does not automatically create system alignment: CHTs tended to prioritize local needs and voices. The statewide priorities are less important to CHTs, which have excellent internal alignment. This creates potential disconnection between state and community health goals. However, CHTs and the VAPM prioritize similar populations, indicating an opportunity to increase alignment by allowing flexible programs tailored to local needs. CHTs also prioritized programs with a strong evidence base, suggesting another potential avenue to create system alignment.

**What is known on this topic**

- Community health teams (CHTs) are a cost‐effective approach to improving community health.
- CHTs are intended to work with the Vermont all‐payer model (VAPM) health reform although the CHTs in Vermont preceded the VAPM.
- The VAPM has led to reductions in healthcare utilization, particularly for acute care stays at the ACO and state levels, as well as a reduction in 30‐day readmissions at the state level in the first 3 years after implementation.

**What this study adds**

- Little is known about how community‐based CHTs set priorities for what social, public health, and medical services to offer.
- Although the VAPM and the CHTs are intended to be cooperative, the alignment between the goals of the VAPM and the priorities of the CHTs is unknown.
- This study adds a better understanding of the preferences and priorities of decision‐makers within CHTs and whether these decisions are aligned with the goals and ambitions set for the VAPM.

## 1 INTRODUCTION

The state of Vermont received a statewide waiver from the Centers for Medicare & Medicaid Services Innovation Center in 2016 to allow all‐payer Accountable Care Organizations (ACOs). A recent preliminary analysis of the overall waiver was favorable, with the independent evaluation finding that Medicare saw gross spending reductions at the state and ACO levels, as well as all‐payer net spending reductions at the state level.^1^ The intermediary evaluation also reported that there were reductions in acute care stays at the ACO and state levels and a reduction in 30‐day readmissions at the state level.^1^

There were some areas where the Vermont all‐payer model (VAPM) has been less successful, however, with enrollment targets being particularly notable.^2^ The initial plan called for 90% of the state population to join within the first 5 years; the actual enrollment has been nearer 50%.^2^ The evaluation also found differential provider participation, with all but one eligible hospitals participating in the Medicaid ACO initiative, but only half of eligible hospitals participating in the Medicare ACO initiative. Notably, most critical access hospitals in Vermont's rural areas opted to not participate in the Medicare ACO initiative.^1^ The most cited barrier to participation was the organizational financial reserves required for the Medicare ACO initiative. This barrier to participation is particularly relevant for smaller organizations providing care to patients and highlights the challenges in creating a statewide reform that works for a heterogeneous landscape of providers and patients.

A continuation of the initial 2016 agreement with the Innovation Center was contingent on achieving the cost, outcome, and participation goals. The waiver, now known as Global Commitment to Health, has been extended by federal regulators to December 2027. Vermont, in many ways, has the optimal conditions for reform because of its unique geographic, physical, political, and other state‐specific attributes.^2^ Other states have also experimented with a (partial) all payer model (APM). Maryland, for example, received an APM for hospitals in 2014, and an evaluation of the waiver provided modest evidence that the APM was succeeding in reducing hospital expenditures without shifting costs to other parts of the healthcare system outside of the global budgets or resulting in adverse impacts on patient outcomes.^3^, ^4^ The rationale for extending the VAPM may be that if the APM works in Vermont, there is no guarantee it will work in other places. However, if does not work in Vermont, it will likely not work elsewhere. The success of the VAPM potentially provides a model for other states; however, replication of the VAPM requires not only replicating the payment model but also other elements of health system reform that preceded the VAPM.

The VAPM is layered upon previous policies that established regional community health teams (CHTs) and medical homes.^1^ The waiver is intended to incentivize a focus on healthcare value and quality and create alignment between the health system payers, providers, and other elements of the care delivery system, including the CHTs, which are not part of the VAPM. However, the extent to which the VAPM created system alignment with the other health delivery system elements, including the CHTs, is unknown.

### 1.1 Community health teams

The regional CHTs have existed for almost two decades with a mission to improve population health statewide. Although CHTs are widely used in many communities and settings, Vermont uniquely deployed CHTs statewide in conjunction with the establishment of Primary Care Medical Homes for all residents. CHTs are a key tool to improve health, control healthcare spending, and create greater health equity.

The statewide network of regional CHTs is multi‐disciplinary, with regional headquarters in each service area's central hospital or Federally Qualified health center. There is at least one CHT in each of the state's 13 Health Service Areas (HSAs) to provide support services for the population of patients and connect individuals with each region's available resources.

The 13 HSAs serve anywhere from 7048 to 93,210 practice‐attributed patients—resulting in between 872 and 15,753 unique annual CHT encounters, respectively.^4^ Staffing includes social workers, dieticians, nurses, and mental health counselors, among others. The CHTs are funded by Medicaid, Medicare, and commercial payers via a pass‐through directed by the Vermont Blueprint for Health initiative (“Blueprint”). This statewide care transformation model emphasizes community‐led strategies for improving health and well‐being. Blueprint ensures that there is at least one CHT in each of the state's 13 HSAs to provide support services for the population of patients and connect individuals with each region's available resources. Payers contribute to the CHT programs through the Blueprint, which then funds the CHTs based on need rather than eligibility. This is in contrast to the VAPM, which focuses on attributed lives (approximately half the state's population), which means they only fund services to the subset of the population that is attributed to the ACO.^5^

The HSAs are divided roughly along county lines and are tasked with improving the health outcomes, health equity, and support systems available in their communities (especially for vulnerable populations) using the funding and resources directed by Blueprint. CHTs work to connect patients to community‐based services, support learning collaboratives, work with medical and community providers to align statewide initiatives with the region's available resources and priorities and improve the quality of services for health and well‐being. The CHTs enable access to individual care coordination, substance use disorder treatment, dietary and nutrition services, and counseling, among other social and economic services.

### 1.2 Interaction between VAPM and CHTs

The VAPM allows value‐based payment incentives across all payers in an attempt to replace the traditional, volume‐based healthcare delivery system with one that rewards the provision of value to patients.^6^, ^7^, ^8^, ^9^ The goal of the VAPM is to shift the entire state from a fee‐for‐service payment model to a value‐based reimbursement model, with targets for spending growth, population health, and care quality. The five‐year targets of the VAPM are ambitious: no more than 3.5% annual cost growth across all payers, 70.0% population enrollment into the model—90.0% for Medicare—and improved health outcomes ranging from lowered suicide rates to reductions in chronic disease incidence. The VAPM's most important priority is thus high‐cost patients.^2^

The VAPM relies on CHTs to help achieve its health system goals, yet does not hire, employ, or control the CHTs directly. It is based on the premise of alignment of performance‐based financing in health with a government budget, which has been applied in other countries.^10^ In theory, the VAPM should create an alignment of incentives for population health while also providing additional funding for expansion of CHTs. Yet the VAPM has a series of statewide goals, in contrast to the community focus of the CHTs.

CHTs gather and consult data about community needs and utilization of services to set priorities for programming and staffing decisions. The Blueprint has an important task in feeding the CHTs relevant data about changing demographics, environmental circumstances, funding mechanisms, and other relevant factors to inform their decision‐making.^5^ The services offered by CHTs are needed in all communities; however, the relative priority of those needs and thus the funding, staffing requirements, and community partnerships required to fully support them vary widely between HSAs. CHTs constantly review and change regional priorities.

Interviewees in our qualitative research described how this strategy cultivates trusting relationships with patients and is an essential step to increase patient engagement for behavioral health modifications. A care coordinator describes their approach as “engaging them [the patient] with what they feel is most meaningful to them at the time. What they need in that moment to see the value to working with someone to get support.” CHTs summarize these needs, which then inform staffing decisions, such as which type of credentialed staff are in highest demand. With the waiver, CHTs are relied upon to both achieve the goals of the ACO and fulfill their public health role, which creates potential tension between the priorities of centralized payers and community stakeholders.

How a global payment mechanism such as VAPM impacts the priorities and design of community‐based interventions such as CHTs is unknown. Indeed, little is known about how community‐based CHTs set priorities for what social, public health, and medical services to offer. In this paper, we examine how community‐based CHTs make trade‐offs made between health, health equity, and healthcare spending.

## 2 METHODS

### 2.1 Experimental design

To understand CHT preferences, we developed a discrete choice experiment (DCE).^11^ The purpose of the DCE is to identify and measure the preferences of the CHTs, which may or may not reflect the priorities of the VAPM. DCEs are commonly used to measure preferences in circumstances where revealed preference data are not readily available.^12^, ^13^ One of the challenges when measuring preferences with a DCE is to develop choice tasks that accurately reflect a real‐life situation.^14^, ^15^ The design of this DCE was based on qualitative research among the program managers and other decision‐makers in the CHTs. In preliminary work, 13 semi‐structured interviews were conducted with program managers from every CHT in the state.^16^ Interviewees were asked about their current service offerings, factors in their decision‐making process, if their decisions were data‐driven, what types of data were consulted, and decision‐making processes.

Interview recordings were transcribed and imported into qualitative research management software. Through processive cycles of coding, the recurring patterns across congruent codes informed the subthemes and these were closely scrutinized for alignment to ensure they were representative of the data. Analysis began at the individual HSA level to establish the context and characteristics of each region, followed by an analysis across all HSAs. Project team members and partners provided feedback on several iterations of emerging categorizations to collapse and refine themes.^5^

Findings were used to define choice attributes and levels in the DCE that resemble real‐life options when choosing between different programs to fund. The interviews identified four themes representing the key priorities for the CHTs: (1) stable and flexible funding, (2) commitment of high‐quality care individualized care coordination that is responsive to the needs of the population, (3) the ability to leverage community partnerships and local resources, and (4) availability of data to support decision‐making. Based on these priorities, five attributes were constructed: cost (Theme 1), the target population (Theme 2), the program champion (Theme 3), and community health plan support (Theme 3) and the availability of data (Theme 4). Theme 3 had two different attributes because program champions and community health plans were separately discussed by the CHTs. Target population was further subdivided into the target population size and effect, and the population affected.

Once the attributes are identified, the next step is to define the different levels of each attribute that define the choice alternatives. The levels need to provide sufficient variation for estimation while also reflecting realistic options. In our study, the levels were defined through a combination of the interview responses and expert opinion. We examined the qualitative interviews for specific examples of levels, such as identifying particularly important target populations. When we did not find specific examples, levels were defined by expert opinion, which was then pilot tested.

For population affected, the survey included six potential groups that could be impacted by the program: the general population; racial and ethnic minorities; persons experiencing homelessness; economically disadvantaged populations; persons with severe chronic health conditions; and persons with substance use disorders. These levels were based on the qualitative interviews in which they were mentioned as important target populations for health and social programs. Population size and effect includes three options: a small population, large effect (the program impacts a small group of people but makes a large impact within the group, with the provided example being a school‐based suicide prevention program targeting 25 at‐risk students), medium population, medium effect (moderate population and moderate impact, e.g., a school‐based asthma prevention program, impacting 100 students and their families), and large population, small effect (large numbers impacted, but the individual impact was small, e.g., a program to improve the walk‐ability and bike‐ability of a neighborhood).

**FIGURE 1:**
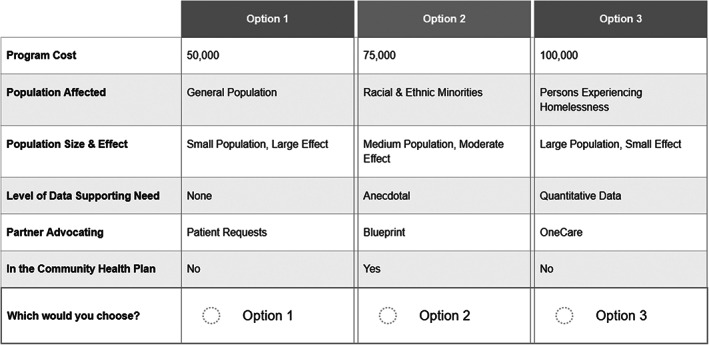
Example choice task.

Potential levels for the data supporting the program were “None,” “Anecdotal,” and “Quantitative Data,” Partners potentially advocating for the program could be patients, primary care providers, local hospitals, a community partner, Blueprint, and OneCare (the only active statewide ACO). Whether the program option would be in the community health plan was binary: Yes or No. For costs, the program options were $50,000; $75,000; and $100,000. Figure 1 shows an example of a choice set which a CHT staff member would have been presented.

There are more than 2000 possible combinations of attributes and levels. We used an iterative computer search to identify a D‐efficient design and eliminate dominant choices.^17^, ^18^, ^19^ In a D‐efficient design, the standard errors are the roots of the diagonal elements of the covariance matrix; the D‐efficient design minimizes the determinant of the covariance matrix which minimizes the standard errors. We used a subsample of the study population as a pilot DCE to estimate “parameter priors,” which are best guesses of the betas and based upon expectations about estimated model parameters. We specified utility functions that include these “priors,” and these can be used to determine the logit probabilities and the log‐likelihood functions. No changes in the DCE design were made between the pilot and the full sample. Software program NGene did the calculations and created the efficient design for the DCE.

Quality checks were performed to identify inconsistent patterns: these included evaluating for invariance in choices (always selecting an option that is in a certain physical position in the choice task), speeding through the survey, and entering random words in free‐text fields.

### 2.2 Survey process

The survey was sent to all CHTs in the state of Vermont via email. Prior to the survey being sent, an introduction to the project was sent by Blueprint encouraging participation. The survey included both CHT leadership and CHT team members. Each choice task included three possible responses with the six attributes. Each respondent was asked to complete the choice task 14 times (with different combinations of choices each time). All respondents completed the same 14 choice tasks.

The survey began by asking sociodemographic questions, followed by the choice tasks, and then several questions about ignored attributes and preference order. For sociodemographics, respondents were asked their gender, age, years of experience, professional background, control of priority setting, and control of funding allocations.

For the choice task, respondents were asked to imagine being in the position of having to decide about the next program to fund for their CHT. The vignette was described:

For the purpose of this study, suppose you are in the position to decide the next program for your team. You are receiving $100,000 in new funding for one of three new programs, which vary in cost. All the programs are equal in terms of administrative complexity, and each of the programs has evidence for their effectiveness. Any funds left over at the completion of the program may be kept by your CHT to support other programs. The new programs differ in the following ways:

After this vignette, the attributes and levels were carefully explained and respondents were asked to proceed to the choice questions and, in every question, to pick the project option that they preferred. A description can be found in the Appendix S1.

### 2.3 Analytic approach

We used conditional logit models^20^ to model the choice as a function of the choice's characteristics (attributes) and mixed multinomial logit (MMNL) to allow distributions for each random (taste) coefficient.^21^, ^22^ Mixed logit models make it feasible to derive individual‐specific estimates conditional on the observed individual choices.^22^, ^23^ The model assumes a specific distribution for each random coefficient, and distributions can vary across the coefficients.

In our model, expected overall utility *U* of respondent *i* from program *j* at the *c*th choice situation is given by

$$U_{\mathrm{ijc}}=\begin{array}{l} \beta_{1i}{\text{Cost}}_{\mathrm{ijc}}+\beta_{2i}{\text{PopulationAffected}}_{\mathrm{ijc}}\\ +\;\beta_{3i}\text{PopulationSizeEffect}+\beta_{4i}{\text{LevelDataSupportingProgram}}_{\mathrm{ijc}}\\ +\;\beta_{5i}{\text{Partner Advocating}}_{\mathrm{ijc}}+\beta_{6i}{\text{InCommunityHealthPlan}}_{\mathrm{ijc}}+\varepsilon_{\mathrm{ijc}}. \end{array}$$

The MMNL model estimates the probability of the observed sequence of choices where its choice probability is estimated using a density function of *β* for which different distributional assumptions can be used. The cost parameter was fixed, and all other parameters were random for which we assumed a normal distribution and used 200 Halton draws. The different attributes were parameterized using dummy variables.

Modeling data from a DCE may also involve including interactions of choice attributes and consumer characteristics. These interactions can be used to test to what extent differences in preferences can be explained by differences in the observed characteristics of the respondents. We developed a number of hypotheses about coefficients varying across groups. We hypothesized that both “patient facing” and “years of experience” would be positively associated with smaller population, larger effect for the population affected (e.g., patient‐facing respondents would prefer programs that had a larger effect for smaller populations).

We also hypothesized preferences would vary by community‐level factors within the HSA the CHT member worked. We hypothesized there would be significant interactions between the average rates of emergency department use and having the local hospital as a program champion; area income and population affected (homelessness and economically disadvantaged); and population health status measures (prevalence of diabetes, cancer, obesity, heart failure, etc.) and severe chronic health conditions. All statistical analysis was performed in Stata 18.

## 3 RESULTS

### 3.1 Descriptive statistics

There were 60 respondents in the survey who completed the 14 choice tasks, with three program options in each task, for a total sample size of 2520. On average, respondents took 34.5 min to finish the survey (standard deviation of 44.9 min). This is more than respondents typically spend on similar choice surveys, suggesting that respondents carefully reviewed the questions and answers. There were respondents from all 13 CHTs, with the most prominent representation from Barre (15 respondents), where a large team participates in the decision‐making process.

Table 1 shows the demographics of the sample. Our sample was 88% female and 12% male. The average age was 47.1 years (standard deviation of 12.5 years), and average years of experience in their current position was 5.3 (standard deviation 5.1 years). Two of the respondents had the official title “program manager” and seven were “CHT Lead,” terms we learned during the qualitative phase of the study have a similar meaning. The remaining respondents were community health workers, nurses, and other staff in the CHT that were part of the decision‐making process regarding priority setting.

**TABLE 1:** Demographics of community health team (CHT) decision‐makers.

Variable	*N*	%	Mean	Std. dev
Gender identity				
Male	7	11.67		
Female	53	88.33		
CHT role				
Program Manager	2	3.33		
Community Health Team Lead	7	11.67		
Community Health Worker	14	23.33		
Self‐Management Educator	4	6.67		
Panel Manager	1	1.67		
Nurse	13	21.67		
Behavioral Health Clinician	6	10.00		
Other—Patient‐facing	11	18.33		
Other—Non‐patient‐facing	2	3.33		
HSA				
Barre	15	25.00		
Bennington	3	5.00		
Brattleboro	3	5.00		
Burlington	6	10.00		
Middlebury	5	8.33		
Morrisville	5	8.33		
Newport	2	3.33		
Randolph	3	5.00		
Rutland	4	6.67		
Springfield	3	5.00		
St. Albans	5	8.33		
St. Johnsbury	4	6.67		
Windsor	2	3.33		
Age (years)			47.07	12.49
Experience (years)			5.27	5.09
Control of priority setting (1–5)^a^			2.73	1.10
Control of funding allocation (1–5)			1.70	0.87
Survey duration (minutes)			34.5	44.9

Abbreviation: HSA, Health Service Area.

^a^ We used a Likert scale from 1 (strongly disagree)–5 (strongly agree).

Respondents were asked for their agreement on a Likert scale from 1 (strongly disagree)–5 (strongly agree) with the statements “I am involved in priority setting for my CHT” and “I am involved in funding allocation for my CHT.” On average, respondents leaned slightly toward disagreeing that they felt involved in priority setting for the CHT (2.73 out of 5, with a standard deviation of 1.10), which reflects the fact that in numbers, there were more community health workers than Team Leads. Since the CHT leadership said they usually include some key community health workers in the decision‐making process, we decided to broaden the scope of respondents. However, this result suggests that they were being consulted but did not necessarily decide about actual resource allocation. This is consistent with the finding that most respondents did not feel in control of funding allocation (1.70 out of 5, with a standard deviation of 0.87).

### 3.2 Mixed logit models

Table 2 shows the results of the base mixed logit model. In the model, all of the attributes were statistically significant except cost. For population affected, programs impacting racial and ethnic minorities were less likely to be selected (reference group: general population), but no other population group was significantly different. Programs targeting a “Large Population with a Small Effect” (reference group: small population, large effect) were also less likely to be selected. Programs were more likely to be selected if they were supported by quantitative data (reference group: no data) and were in the community health plan. Programs advocated for by the all‐payer ACO were also less likely to be selected.

**TABLE 2:** Results of mixed logit model showing the effect of attributes on the probability of choices.

	Coefficient	Std. error	95% CI	*p*‐value
Cost ($1000s)	0.018	0.026	[−0.033, 0.070]	0.490
Population affected				
General population [Ref.]	‐			
Racial and ethnic minorities	−0.631**	0.261	[−1.142, −0.119]	0.016
Persons experiencing homelessness	0.313	0.286	[−0.249, 0.874]	0.275
Economically disadvantaged	0.382	0.238	[−0.085, 0.849]	0.109
Persons with severe chronic health conditions	−0.021	0.259	[−0.530, 0.487]	0.934
Persons with substance use disorder	0.213	0.252	[−0.280, 0.706]	0.397
Population size and effect				
Small population, large effect [Ref.]	‐			
Medium population, moderate effect	0.070	0.121	[−0.167, 0.307]	0.562
Large population, small effect	−1.864***	0.286	[−2.425, −1.303]	0.000
Level of data supporting need				
None [Ref.]	‐			
Anecdotal	0.073	0.149	[−0.218, 0.364]	0.623
Quantitative data	0.919***	0.181	[0.566, 1.273]	0.000
Partner advocating				
Blueprint [Ref.]	‐			
Patient requests	−0.164	0.231	[−0.616, 0.288]	0.477
OneCare	−0.457*	0.248	[−0.943, 0.028]	0.065
Local primary care	0.152	0.222	[−0.284, 0.587]	0.494
Local hospitals	0.271	0.226	[−0.172, 0.714]	0.230
Community partner	−0.061	0.212	[−0.477, 0.355]	0.773
In community health plan?				
No [Ref.]	‐			
In community health plan	0.705***	0.147	[0.417, 0.994]	0.000
ASC (Program 1)	−0.063	0.131	[−0.320, 0.193]	0.630
ASC (Program 3)	−0.053	0.131	[−0.310, 0.205]	0.690
Observations	2520			
Individuals	60			
AIC/BIC	1497/1653			
Log likelihood	−715.7			

*** *p* < 0.01;

** *p* < 0.05;

* *p* < 0.1.

In all cases, the coefficients were invariant across the different respondent groups and HSAs. This does not necessarily mean there is no preference heterogeneity among different subgroups of our sample, but we may simply not have been able to capture it with our relatively small sample size.

### 3.3 Attribute importance

**FIGURE 2:**
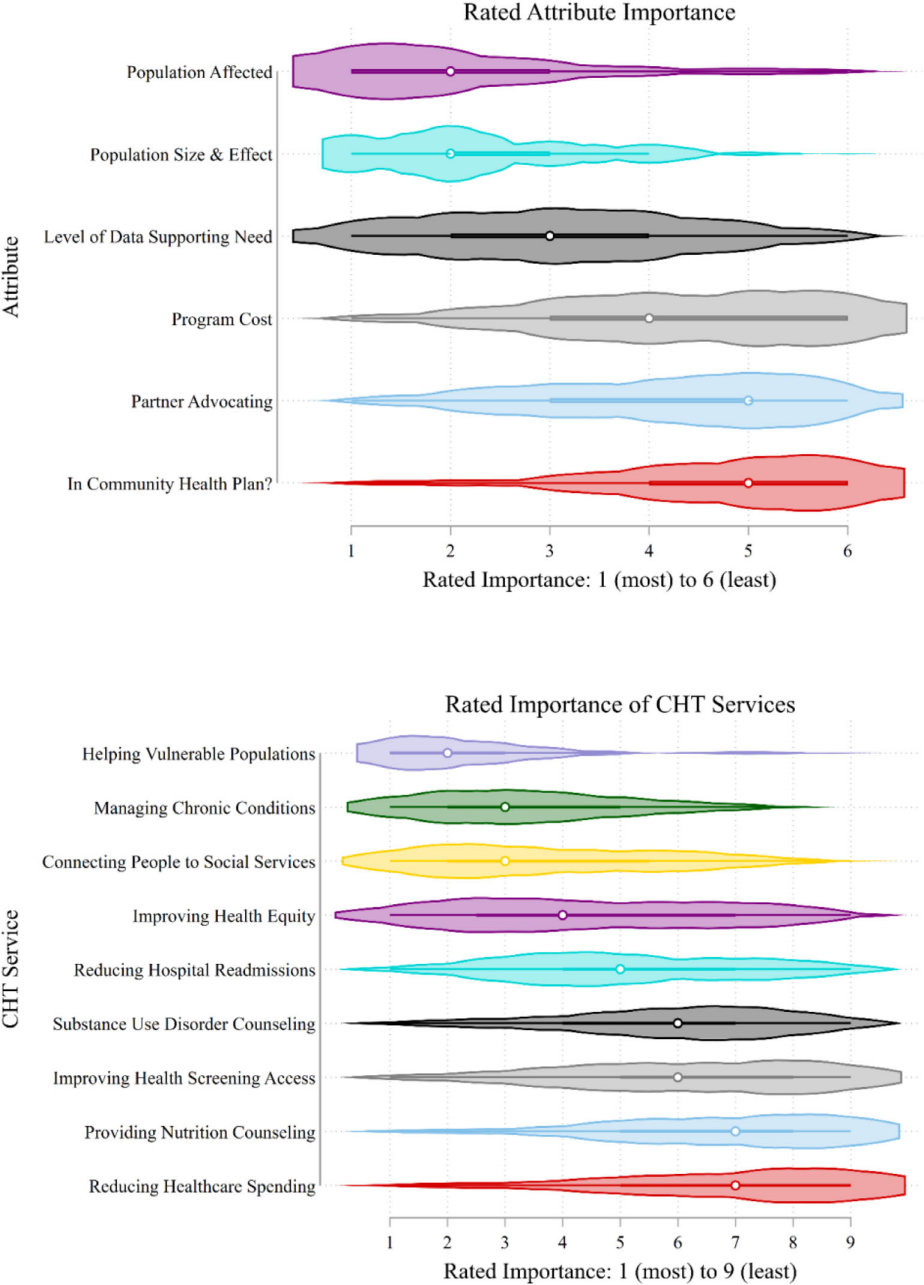
Violin plot showing rated attribute importance by survey respondents.

We also asked questions regarding the relative importance of the different attributes by asking respondents to list their most important attributes in order of preference (Figure 2). These normative questions showed a pattern consistent with the results of the logit models. The figure shows two violin plots, which is a statistical graphic showing the probability density of the data at different values, so we can conclude how much agreement there is among respondents. The ranking is 1 for most important and 6 for least important. The first plot shows the answers to the question “Please rank the importance of the various factors you used to make your decisions, from most important, to least important.” Most respondents indicated that they cared the most about the population that a new program would affect and the population's size and effect. They also indicated that the need to invest in a particular population would have to be supported by the data. Respondents placed a lower value on the program's cost, whether partners would be advocating the program and whether the program would be in the community health plan.

Figure 2 also shows the answers to the question “When you think about the following services offered by your Community Health Team, how would you rank them from most important, to least important?” The options included other attributes of programs that the respondents could prioritize, such as reducing healthcare spending, reducing hospital readmission, helping vulnerable populations, improving health equity, connecting people to social services, helping people manage their chronic health conditions, providing nutrition counseling, providing substance use counseling, and improving access to health screenings. The top priorities were that it would help vulnerable populations, assist with managing chronic conditions, and connect people to social services. The fourth most important attribute of services would be improving health equity, followed by reducing hospital admissions. Even though some CHTs provide targeted help for people with substance abuse disorders, health screening access, and nutrition counseling, these were not given a high priority by the different decision‐makers in the CHTs. These relative attribute importance measures resemble the utility weights in the mixed logit model.

Some CHTs reported they offer patient‐centered services, meaning that service offerings would be based on what patients indicated they needed help with. However, this is not reflected in the relative importance of attributes of programs they can offer. In other words, these services may be offered at the individual patient level but do not affect decisions for resource allocation and priority setting for population‐based programs. The least important attribute for respondents was a reduction in healthcare spending.

### 3.4 Alignment within CHTs

Lastly, we tested whether there was a difference in priorities between CHT leadership and CHT staff in terms of ranked attribute importance. In Table 3, we show median ranking of the attribute importance from 1 (very important) to 6 (not important) by the staff and the leadership. Table 3 shows the attribute importance by CHT role, which illustrated there was general agreement about priorities, where leadership slightly favored costs (5 vs. 4) and staff slightly favored population affected (2 vs. 1).

**TABLE 3:** Ranking of attribute importance, stratified by community health team (CHT) role*.

	CHT role			
	Staff	Leadership	Total	*p*‐value
	Median (SD)	Median (SD)	Median (SD)	
In community health plan?	5 (1.48)	5 (1.30)	5 (1.46)	0.834
Partner advocating	5 (1.43)	5 (0.86)	5 (1.36)	0.668
Program cost	4 (1.43)	6 (1.27)	4.5 (1.44)	0.093
Level of data supporting need	3 (1.50)	3 (1.05)	3 (1.44)	0.592
Population size and effect	2 (1.34)	2 (1.11)	2 (1.31)	0.763
Population affected	2 (1.56)	1 (0.71)	2 (1.49)	0.140

*Note*: Ranking on a 1 to 6 scale, with 1 ranked as “highest priority” and 6 as “lowest priority.”

## 4 DISCUSSION

In this paper, we examined CHT's preferences for health, equity, and spending considerations when making decisions about resource allocation and the alignment of these preferences with VAPM priorities. We found that CHTs prioritized programs in the community health plan and those with quantitative evidence of effectiveness. They were less likely to choose either programs targeting racial and ethnic minorities or programs having a small effect on a large population. Preferences did not vary across individual or community attributes. Program priorities of the VAPM, especially healthcare spending, were not prioritized.

The VAPM represents a model for funding both healthcare and CHTs that is potentially replicable in other states and other countries. Understanding how the VAPM impacts the CHTs thus provides lessons for other states as they design models to support CHTs. However, in this model, decision‐makers from CHTs are not required to prioritize programs that are prioritized by the VAPM. In addition, indeed, we found that CHTs did not necessarily prioritize programs championed by the VAPM through the ACO. Instead, we found that CHTs prioritized programs that were in the community health plan and programs with quantitative evidence of effectiveness.

The results were generally consistent with our prior hypotheses. The importance and need for data supporting program effectiveness matches the qualitative interviews, as does the emphasis on smaller populations and large impacts and the importance of the community health plans, which were often developed with strong input from the CHTs. The negative coefficient on Racial and Ethnic Minorities was surprising and potentially warrants further study. It could reflect the lack of diversity in Vermont or a desire by the CHTs to focus on health and economic issues rather than race and ethnicity. The mixed model results included some fairly large effects that were not statistically significant, and we did not find significant interaction effects even though one might expect some preference heterogeneity within CHTs. Our sample size was relatively small, and it is possible we could have been able to detect some of these effects more accurately with a larger sample size. Future research should focus on expanding this study among community health workers in other states, which may also allow to compare and contrast between states.

The results suggest that the new VAPM does not automatically create system alignment: the CHTs tended to prioritize local needs and local voices. The statewide priorities are less important to the CHTs, which have excellent internal alignment. This creates a potential disconnection between the state and community health system goals. However, the CHTs do prioritize the populations that are also prioritized by the VAPM—programs that have large health impacts on small populations will also likely impact the high‐cost populations prioritized by the VAPM—indicating an opportunity to increase alignment by allowing for more flexible implementation of programs, which allow for tailoring to local community needs.

Our results suggest a roadmap to create alignment. First, there is a need to create consistency between the statewide goals and the community health plans. The ACO and the state are focused on the broad statewide goals, but the CHTs are working with local partners to create and implement local community health plans. Aligning the statewide goals and the community health plans could help alignment of top‐down versus bottom‐up priorities.

Secondly, there is a need for commonality between covered populations. The VAPM only covers approximately half of the population of the state; meanwhile, CHT services are offered to anyone in need in the community. As the VAPM expands enrollment, some of this tension will be resolved although this expansion is heavily dependent on political will and the shifting insurance landscape.

Finally, the CHTs want to prioritize programs that have proven effectiveness supported by quantitative data. This suggests that collecting data on the effectiveness of programs and then providing the data to CHTs would be an effective way to create alignment. In previous work, a few CHTs mentioned they are creating their own data reporting systems and dashboards to inform their decision‐making and communicate progress.^16^ One CHT team shared that they created a quality dashboard to present at meetings with community partners reviewing data related to diabetes, hypertension, food insecurity, and substance use disorder. Some program managers shared they would like to consult data more consistently when making decisions but lack the resources and capacity. An evidence‐based approach to program prioritization would also allow resources to be targeted toward programs that are more likely to have a significant impact on the health of the population.

## CONFLICT OF INTEREST STATEMENT

The authors declare no conflicts of interest.

## Supporting information


**Appendix S1.** Supporting information.
