# Supplementary material for: Examining alignment of community health teams' preferences for health, equity, and spending with state all‐payer waiver priorities: A discrete choice experiment
Source: Health Serv Res. 2023 Nov 14;59(Suppl 1):e14257. doi: 10.1111/1475-6773.14257 (PMC10796287; doi:10.1111/1475-6773.14257)
Supplement: Supplementary file 1 — Appendix S1. Supporting information. [file HESR-59-e14257-s001.docx]

**Appendix: Attributes and Levels in Discrete Choice Experiment**

For the purpose of this study, suppose you are in the position to decide the next program for your team.You are receiving $100,000 in new funding for one of three new programs, which vary in cost.  All the programs are equal in terms of administrative complexity, and each of the programs has strong evidence for their effectiveness. Any funds left over at the completion of the program may be kept by your CHT to support other programs. The new programs differ in the following ways:

- Program Cost. This is the amount of money (USD) you would need to spend to run the program, the left-overs of which your team would be allowed to keep. Options you will be presented with include:
  1. *$50,000*- This means that you would need to spend $50,000 to implement the program, and would have $50,000 left over.
  2. *$75,000*- This means that you would need to spend $75,000 to implement the program, and would have $25,000 left over.
  3. *$100,000* - This means that you would need to spend $100,000 to implement the program, and would have $0 left over.
- Population Affected. Some programs are targeted at particular vulnerable populations in the community. This is the population in your community which the program is designed to benefit, and who would gain the most from the program. Options you will be presented with include:
  1. *General Population*- This means that the program would impact the entire population of your HSA equally
  2. *Racial & Ethnic Minorities*- This means that the program would target racial and ethnic minorities in your HSA (e.g., BIPOC, New Americans)
  3. *People Experiencing Homelessness*- This means that the program would target people experiencing homelessness in your HSA.
  4. *Economically Disadvantaged*- This means that the program would target economically disadvantaged individuals in your HSA.
  5. *People with Severe Chronic Health Conditions*- This means that the program would target people with severe chronic health conditions in your HSA.
- Population Size and Effect. This is the size of the population in your community which will receive a health benefit from the program, and how much they will be affected. Options you will be presented with include:
  1. *Small Population, Large Effect* - This means that the program will impact a small vulnerable population, but each impacted person will benefit greatly. An example of such a program could be a school suicide prevention program, impacting 25 at-risk students.
  2. *Medium Population, Moderate Effect* - This means the program will impact a larger number of people, and each person will receive some direct benefit. An example of such a program could be a school-based asthma prevention program, impacting 100 students and their families.
  3. *Large Population, Small Effect* - This means the program will impact a large number of people, but the health gain for each individual will be relatively small. An example of this could be a program to improve the walk-ability and bike-ability of a neighborhood.
- Level of Data Supporting Program. This is the kind of data which is available to support the need for the program in your community. Options you will be presented with include:
  1. *None*- This option means that there is no specific data for or against the program in your community
  2. *Anecdotal*- This means there is information gathered from word-of-mouth in the community to support the program's need / benefit
  3. *Quantitative Data -* This is a numerical, objective measure of the need and potential benefit of the program. Sources could include reports from your local Emergency Department, OneCare app/dashboard data, Blueprint data, or scientific papers.
- Partner Advocating. This is the CHT partner who is most strongly pushing for the selection of the program. Options you will be presented with include:
  1. *Patient Requests*- This means that patients in your HSA have asked for this program
  2. *Blueprint*- This means that Blueprint has identified this program as a priority
  3. *OneCare*- This means that OneCare has identified this program as a priority
  4. *Local Primary Care Practices*- This means that local primary care practices in your HSA have asked for this program
  5. *Local Hospitals*  - This means that local hospitals in your HSA have asked for this program
  6. *Other Community Partner*- This means that a community partner you work with has asked for this program
- In the Community Health Plan? This indicates whether this specific program, and the vulnerable population it affects, is referenced in your HSA's community health plan as an existing need. Options you will be presented with include:
  1. *No*- This means that the program is not specifically listed in your community health plan
  2. *Yes*- This means that the program is specifically referenced as a need in your community health plan
